# Instrumental and Non-Instrumental Evaluation of 4-Meter Walking Speed in Older Individuals

**DOI:** 10.1371/journal.pone.0153583

**Published:** 2016-04-14

**Authors:** Marcello Maggio, Gian Paolo Ceda, Andrea Ticinesi, Francesca De Vita, Giovanni Gelmini, Cosimo Costantino, Tiziana Meschi, Reto W. Kressig, Matteo Cesari, Massimo Fabi, Fulvio Lauretani

**Affiliations:** 1 Geriatric Rehabilitation Department, University-Hospital of Parma, Parma, Italy; 2 Department of Clinical and Experimental Medicine, University of Parma, Italy; 3 Azienda USL of Parma, Distretto Valli di Taro e Ceno, Parma, Italy; 4 Memory Clinic, University Center for Medicine of Aging Basel (UAB), Felix-Platter Hospital, University of Basel, Switzerland; 5 Gérontopôle, Centre Hospitalier Universitaire de Toulouse, Toulouse, France; 6 Université de Toulouse III Paul Sabatier, Toulouse, France; 7 General Direction, University-Hospital of Parma, Italy; University of Brescia, ITALY

## Abstract

**Background:**

Manual measurement of 4-meter gait speed by a stopwatch is the gold standard test for functional assessment in older adults. However, the accuracy of this technique may be biased by several factors, including intra- and inter-operator variability. Instrumental techniques of measurement using accelerometers may have a higher accuracy. Studies addressing the concordance between these two techniques are missing. The aim of the present community-based observational study was to compare manual and instrumental measurements of 4-meter gait speed in older individuals and to assess their relationship with other indicators of physical performance.

**Methods:**

One-hundred seventy-two (69 men, 103 women) non-disabled community-dwellers aged ≥65 years were enrolled. They underwent a comprehensive geriatric assessment including physical function by Short Physical Performance Battery (SPPB), hand grip strength, and 6-minute walking test (6MWT). Timed usual walking speed on a 4-meter course was assessed by using both a stopwatch (4-meter manual measurement, 4-MM) and a tri-axial accelerometer (4-meter automatic measurement, 4-MA). Correlations between these performance measures were evaluated separately in men and women by partial correlation coefficients.

**Results:**

In both genders, 4-MA was associated with 4-MM (men r = 0.62, p<0.001; women r = 0.73, p<0.001), handgrip strength (men r = 0.40, p = 0.005; women r = 0.29, p = 0.001) and 6MWT (men r = 0.50, p = 0.0004; women r = 0.22, p = 0.048). 4-MM was associated with handgrip strength and 6MWT in both men and women. Considering gait speed <0.6 m/s as diagnostic of dismobility syndrome, the two methods of assessment disagreed, with a different categorization of subjects, in 19% of men and 23% of women. The use of accelerometer resulted in 29 (13 M, 16 F) additional diagnoses of dismobility, compared with the 4-MM.

**Conclusions:**

In an older population, the concordance of gait speeds manually or instrumentally assessed is not optimal. The results suggest that manual measures might lead to misclassification of a substantial number of subjects. However, longitudinal studies using standardized and validated procedures aimed at the comparison of different techniques are needed before recommending the use of accelerometers in comprehensive geriatric assessment.

## Introduction

Gait speed, or *walking speed*, measured at the individual’s usual pace has been reported to be a relevant clinical marker of health, well-being and functional status of older population [1]. Epidemiological studies addressing the reliability and validity of gait speed assessment in this age-group indicate that this parameter is an independent predictor of a wide range of poor clinical outcomes in older persons, including falls [2], hospitalization/institutionalization [3], disability [4] and mortality [5].

In particular, the 4-meter gait speed test is one of the most widely used assessment tools in the clinical geriatric settings [6]. This measurement, performed by a stopwatch, is simple, quick, reproducible, inexpensive, feasible, and can even be assessed by non-professional trained staff.

Recent recommendations from the *Mobility Working Group* have identified the timed 4-meter usual gait speed as the main tool to diagnose dismobility, a condition characterized by poor mobility and defined by gait speed slower than 0.6 m/s [7–8].

However, the current standard methodology of assessment of the walking time during the test by a stopwatch can be biased by high inter- and intra-operator variability [9,10]. These limitations might persist even after an intensive training for the staff devoted to its use. Over the past two decades, the increasing need of accurate and objective techniques in the assessment of physical activity led to the technological development of inexpensive, miniature accelerometer sensors [11,12]. The quality of the information provided by these sensors is potentially more reliable and valid and may theoretically give better quantitative measures of gait in older individuals, identifying deteriorating gait and dismobility [13].

However, studies investigating the correlation between the assessment of gait speed, when conducted with a traditional stopwatch and by using accelerometers, are missing. Scarce information is also available on the relationship between the results obtained with these two assessment methods and other objective tests of physical performance [14]. In a recent population-based cohort study carried out in older individuals without activities of daily living (ADL) disability, a good correlation between manually-measured 4-meter gait speed and Short Physical Performance Battery (SPPB) was found, but instrumental measurements with accelerometers were not performed [15].

Given the clinical and research relevance of slow gait speed, we sought to investigate the correlation between its manual (using a stopwatch, 4-MM) and technological (using an accelerometer, 4-MA) assessment in older individuals. The study was also aimed at evaluating the correlation of 4-MM and 4-MA with other measures of physical performance, namely hand grip strength and the 6-minute walking test (6MWT).

## Materials and Methods

### Design, Participants and Ethical Statement

This community-based observational study is an ancillary project of the MED&SANO study, an epidemiological study conducted in a representative sample of non-disabled older persons living in the Medesano geographic area (Emilia Romagna Region, Italy). The current analysis used cross-sectional data from the baseline comprehensive geriatric assessment conducted between November 2012 and April 2013 as part of the Provide multicenter European study, whose aims and design are detailed elsewhere [16].

A total of 455 non-disabled older community-dwellers aged 65 years or older were eligible for study enrolment after a screening evaluation by general practitioners of the Medesano area, according to the absence of physical disability. Among them, 205 were randomly selected to undergo a second-level specialist evaluation including comprehensive geriatric assessment. All subjects with overt disability (Barthel index score <65), with severe chronic diseases or cancer were excluded from the study at this second step (n = 8). For the present analysis, 172 participants (69 men and 103 women) with complete data for the measures of interest were considered. Thus, 33 subjects were excluded since they did not complete the battery of physical tests included in this study.

The study protocol was approved by the Ethics Committee of Parma Province. All participants were informed about the study procedures, purposes, and known risks, and all gave their written informed consent.

### Physical Performance and Muscle Strength Measures

The battery of physical evaluation tests included the assessment of gait speed by using a stopwatch (4-MM) and an accelerometer (4-MA), SPPB, 6MWT and hand grip strength. For each participant, all measures were collected during the same day. SPPB was assessed first, followed, respectively, by 6MWT, 4-meter gait speed and handgrip strength. A period of 2–3 minutes of rest was granted between one test and another.

#### Gait Speed Assessment

The manual and instrumental assessment of gait speed were performed during the same 4-meter walk. A straight clearly marked course was used. The manual measurement was made by a trained operator using a stopwatch (4-MM). A tri-axial accelerometer was instead used for the instrumental assessment (4-MA) [17]. Instructions to walk at usual pace from a still standing position behind the starting line were provided to participants. Timing started at the first foot movement and ended when a foot completely crossed the finish line. Evaluations were conducted in a standardized way by five trained investigators. Canes and walkers were allowed if the subject normally used this equipment in his/her daily life.

The inertial triaxial sensor (Free4Act®, LorAn-Engineering, Bologna, Italy) consists of a small case of 78x48x20 mm weighting 48 g only, easy to use, requires no specialized equipment, does not interfere with regular walking, and could be used to analyze walking in clinical practice. The accelerometer, placed on a semi-elastic belt covering the L4-L5 inter-vertebral space, transmitted the data to a PC via Bluetooth. The sensitive axes of the sensing unit were automatically aligned along the anatomical vertical, medio-lateral, and antero-posterior axis.

### Short Physical Performance Battery (SPPB)

Lower extremity performance was evaluated using the SPPB, a strong predictor of physical disability in older adults [18]. The SPPB includes three timed subtests: the chair stand test, the usual gait speed test, and the balance test. The timed results of the subtests were converted to an ordinal scale ranging from 0 (worst performance) to 12 (best performance) according to predefined and previously published cut-points.

#### 6-minute Walking Test (6MWT)

The 6MWT was performed indoors, using a 30 meter-long walking course with a hard surface. The length of the corridor was marked every 3 meter and cones marked the turnaround points [19]. A starting line, defining the beginning and the end of each 60-meter lap, was marked on the floor using a brightly colored tape. Participants were invited at walking as far as possible over the circuit for a period of 6 minutes. The test evaluates the global and integrated responses of all the systems involved during exercise, including the pulmonary and cardiovascular systems, systemic circulation, peripheral circulation, blood, neuromuscular units, and muscle metabolism [19].

#### Handgrip Strength

Isometric hand grip strength was measured using a handheld dynamometer (Jamar Plus Digital Hand Dynamometer). The device measured strength in kilograms, with a precision of 0.1 kg. Participants were asked to perform the task twice with each hand. The average of the best result obtained with each hand was used for the present analyses.

#### Other Measures

Weight and height, objectively measured and employing a standard protocol, were used to calculate the body mass index as kg/m^2^. The Barthel Index was assessed for measuring the participants' functional ability. Cognitive performance was assessed using the Mini-Mental State Examination (MMSE). Depressive symptoms were assessed by the 15-item Geriatric Depression Scale (GDS-15), which is a widely used screening instrument for depressive symptoms in the elderly. The GDS-15 detects changes in depressive symptoms after a major negative life event [20]. Nutritional status was determined using the Mini Nutritional Assessment–Short Form (MNA-SF), which is a reliable and practical screening test validated in all geriatric settings [21]. Information on drug use was collected through the expert evaluation of clinicians as routine approach.

The prevalence of specific medical conditions was established using standardized criteria that combine information from self-reported history, medical records, and a clinical medical examination. Diagnostic algorithms were modified versions of those adopted in the Women’s Health and Aging Study [22].

### Statistical Analysis

Categorical variables were expressed in numbers and percentages, and continuous variables were reported according to gender as means (and standard deviations, SD) for normally distributed parameters or as median and interquartile range (IQR) for those non-normally distributed.

Since reference values for some muscle function and gait parameters in non-disabled older individuals are different between males and females [23], a gender-specific analysis was carried out. Scatterplots of data were built to examine the correlation between 4-MM and 4-MA. Then, unadjusted Pearson correlations were calculated, as appropriate. Correlations of 4-MM and 4-MA with hand grip strength and 6MWT were also separately assessed in men and women using the same tests. 4-MA and 4-MM were categorized by using the cut-off point for dismobility syndrome of 0.6 m/sec [7,8]. The concordance between 4-MM and 4-MA in diagnosing dismobility syndrome was then assessed.

Statistical significance was defined as p≤0.05. SAS 8.2 statistical package (SAS Institute, Inc., Cary, NC, USA) was used for all analyses.

## Results

Characteristics of the study population (n = 172, 69 M, 103 F) are summarized in Table 1. The mean age of participants was 80.7 (SD 4.8) years.

**Table 1:** Sociodemographic and Clinical Characteristics of Study Sample of the MED&SANO study.

	Men(n = 69)	Women(n = 103)
Age (years)	79.03 ± 4.87	78.18 ± 5.64
Barthel Index	97.78 ± 5.45	96.65 ± 6.47
Handgrip strength (kg)	39.42 ± 8.57	23.85 ± 5.01
Total SPPB (score)	9.74 ± 1.14	8.97 ± 1.65
4-MM (m/s)	0.91 ± 0.26	0.78 ± 0.22
6MWT (m/s)	0.88 ± 0.22	0.86 ± 0.21
4-MA (m/s)	1.06 ± 0.17	0.89 ± 0.18
BMI (kg/m^2^)	26.56 ± 3.18	26.24 ± 3.80
MMSE score	26.13 ± 3.17	26.81 ± 3.55
GDS Short Form (score)	3 [1–5]	3 [2–6]
MNA Short Form (score)	13.14 ± 1.47	12.45 ± 2.07
Drug use (n)	3 [2–5]	4 [3–6]

*Data are presented as number of cases (percentage), mean ± SD or median and interquartile range.

**SPPB; Short Physical Performance Battery; 6MWT: Six Minute Walking Test; 4-MA: 4-m gait speed accelerometer; 4-MM: 4-m gait speed manual; BMI: Body Mass Index; MMSE: Mini Mental State Examination Score; GDS: Geriatric Depression Scale; MNA: Mini Nutritional Assessment.

**Fig 1:**
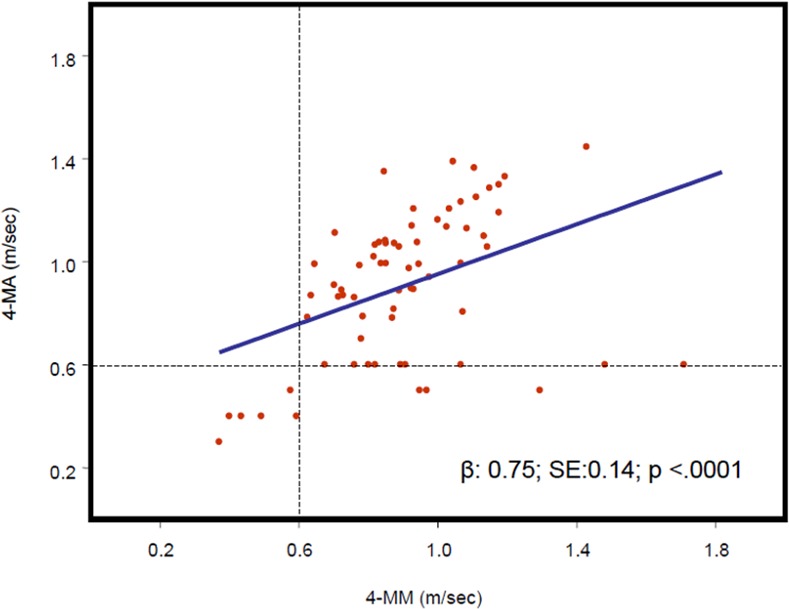
Scatterplot representing 4-MM and 4-MA values in male participants (n = 69). The regression curve and regression equation between 4-MM (horizontal axis) and 4-MA (vertical axis). (4-MA: 4-m gait speed accelerometer; 4-MM: 4-m gait speed manual).

**Fig 2:**
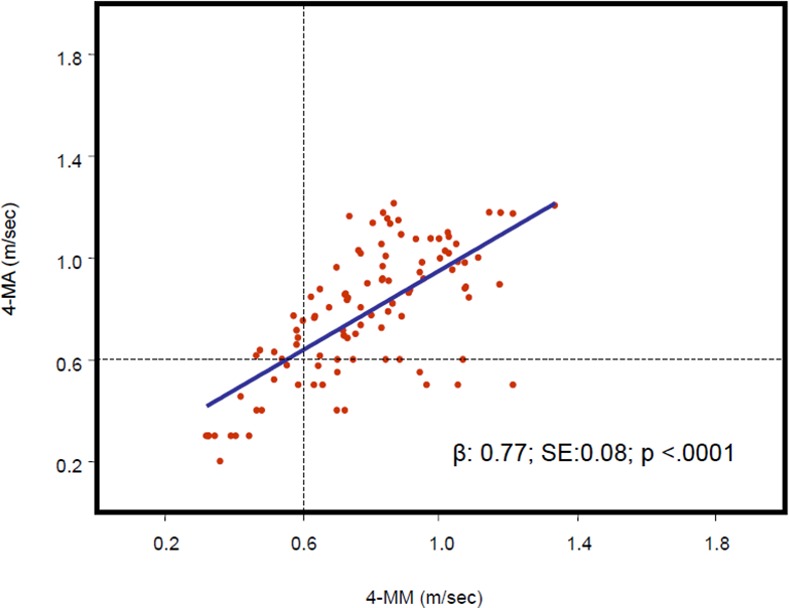
Scatterplots representing 4-MM and 4-MA values in female participants (n = 103). The regression curve and regression equation between 4-MM (horizontal axis) and 4-MA (vertical axis).(4-MA: 4-m gait speed accelerometer; 4-MM: 4-m gait speed manual).

Scatterplots of correlations between 4-MM and 4-MA in men and women are shown in Figs 1 and 2, respectively. In both genders the correlations were statistically significant (β±SE men 0.75±0.14, p<0.001, women 0.77±0.08, p<0.001). Figs 1 and 2 also show the categorization of subjects as having or not having dismobility syndrome according to the cut-off value of 0.6 m/s.

Calculation of unadjusted Pearson correlations confirmed that 4-MM was significantly and positively correlated with 4-MA in both genders, as highlighted in Table 2 (r = 0.62, p<0.001 in men; r = 0.73, p<0.001 in women). Among males, the number of subjects categorized as having dismobility syndrome (gait speed <0.6 m/s) was 6 (9%) according to 4-MM, and 19 (28%) according to 4-MA. Among females, 22 subjects (21%) had dismobility syndrome according to 4-MM and 30 (29%) according to 4-MA. Table 3 shows the categorization of participants according to the different measurement techniques and using 0.6 m/s as gait speed cut-off in men and women, respectively. In 13/69 males (19%) and 24/103 females (23%) the two methods of gait speed assessment disagreed for the presence of dismobility syndrome.

**Table 2:** Unadjusted coefficient correlation investigating the relationship between 4-MM, 4-MA and objectives measures of physical performance and functional capacity.

	Men(N = 69)				Women(N = 103)			
	4-m gait speed				4-m gait speed			
	4-MM		4-MA		4-MM		4-MA	
	r	P	r	P	r	P	r	P
**Handgrip strength**	0.51	< .0001	0.40	0.005	0.38	0.0001	0.29	0.001
**4-MM**	-	-	0.62	< .0001	-	-	0.73	< .0001
**6-MWT**	0.59	< .0001	0.50	0.0004	0.49	< .0001	0.22	0.048
**4-MA**	0.62	< .0001	-	-	0.73	< .0001	-	-

4-MM: 4-meter walking speed measured manually by stopwatch; 4-MA: 4-meter walking speed measured instrumentally by accelerometer; 6MWT: 6-minute walking test.

**Table 3:** Categorization of male (n = 69) and female participants (n = 103) by using gait speed cut-off 0.6 m/s according to the two different methods of gait speed assessment (4-MM, manual assessment; 4-MA accelerometer assessment). 4-MM showed a poor sensitivity to detect dismobility syndrome (32% for males and 47% for females).

	**Males (N = 69)**	
**4-MA4-MM**	<0.6 m/s (dismobility)	≥0.6 m/s (normal)
<0.6 m/s (dismobility)	6 (8.7%)	0
≥0.6 m/s (normal)	13 (18.8%)	50 (72.5%)
	**Females (N = 103)**	
**4-MA4-MM**	<0.6 m/s (dismobility)	≥0.6 m/s (normal)
<0.6 m/s (dismobility)	14 (13.6%)	8 (7.8%)
≥0.6 m/s (normal)	16 (15.5%)	65 (63.1%)

The correlations of 4-MM and 4-MA with other measures of functional performance are also shown in Table 2. A significant positive correlation with 6MWT was found for both 4-MM and 4-MA in men and women (4-MM men r = 0.59, p<0.001; women r = 0.49, p<0.001; 4-MA men r = 0.50, p = 0.0004; women r = 0.22, p = 0.048).

4-MA showed a significant positive correlation with handgrip strength (r = 0.40, p = 0.005 in men; r = 0.29, p = 0.01 in women), as also 4-MM (r = 0.51, p<0.001 in men; r = 0.38, p = 0.0001 in women).

## Discussion

In a cohort of community-dwelling older individuals, we found a significant correlation between the assessment of gait speed using a manual (i.e., stopwatch) and technological (i.e., accelerometer) technique. However, our results suggest that the concordance of two tests is less strong than anticipated and might be suboptimal in the classification of single subjects. This is the first study investigating the correlation between these two assessment modalities of gait speed.

There is a wide range of methods available for assessing physical function in both research and clinical practice [24]. The final choice on the best measurement should take into account the inter-rater and test-retest reliability, accuracy, feasibility and costs. Namely, 4-meter gait speed test was shown to be extremely robust. Moreover, its design makes it particularly suitable for use in routine clinical and research activities. These characteristics led to its integration in the SPPB, the most used objective tool for the assessment of lower extremity functioning in older persons [24].

The present study shows that both 4-MM and 4-MA were correlated to other tests of physical performance like 6MWT and hand grip strength. The correlation found between the 4-MM and the 4-MA highlights that further studies are needed to investigate the role of accelerometer-measured gait speed assessment. Accurate, standardized and reproducible techniques of measurement are in fact needed, privileging those techniques less affected by methodological issues, and inter- and intra-operator variability that may represent a relevant bias [9,10].

Four-meter gait speed depends on lower limb muscle function and is a good predictor of mobility impairment and adverse outcomes [8,25,26]. Thus, it is important to use appropriate, reliable and accurate techniques to measure it during the comprehensive geriatric assessment, in order to correctly diagnose dismobility syndrome. In fact, a 4-meter walking speed lower than 0.6 m/s is nowadays widely recognized as a diagnostic criterion for this syndrome [7]. We acknowledge that this cut-off point was developed using 4-MM and not based on 4-MA. Our data clearly show that a large number of cases were categorized differently according to the method of assessment. By hypothesizing 4-MA as gold standard, 4-MM showed a poor sensitivity to detect dismobility syndrome (32% for males and 47% for females, Table 3). As such, the way gait speed is measured may have strong clinical implications. The use of an accelerometer may represent a promising alternative to manual assessment, theoretically giving more “objective” results. However, the method of wearing the device in 4-MA might influence the gait information obtained from this instrument. In addition, further longitudinal studies should investigate the correlation of 4-MA with functional outcomes, particularly in those subjects categorized differently according to the two techniques. Moreover, these results reinforce the concept that there are some gender-related differences in gait speed, that should be considered when interpreting results of functional tests during a comprehensive geriatric assessment.

4-MM remains the easiest and commonest way to assess physical performance [6]. Nevertheless, when using this technique, a certain degree of variability, potentially influencing its results, should always be taken into account. The routine application of 4-MA may help to overcome these limitations in both clinical and research settings where a high degree of concordance is needed to detect dismobility syndrome,.

The problem of insufficient accuracy of manual measurements of walking speed is well known in medical literature [9,10,27]. Some studies addressed the importance of an initial specific training of the staff to reduce the potential inaccuracy of evaluation [9,10]. Others used a photocell-based method (with devices mounted at the starting and finishing lines) associated with a rigid protocol of administration. As compared to manual methods of assessment, gait-accelerometers provide a wider spectrum of additional information that can be clinically relevant for exploring the risk of functional declines in older subjects (e.g., variability of movements, balance, static and dynamic acceleration). Accelerometers are also characterized by a portable and low cost acquisition system. In addition, this testing is not restricted to a laboratory environment since this instrument easily allows the assessment of usual gait speed in all the settings [26]. Several investigations evaluating measures of gait in subjects with different neurological conditions with pathological gait impairment, compared to healthy subjects, have already suggested that a triaxial accelerometer is a good practical tool for capturing altered ambulation [28].

Our study has some limitations. The relatively small sample size and the cross-sectional nature of the study do not allow definitive conclusions. The capacity of 4-MA to predict functional outcomes in older community-dwellers is in fact still poorly investigated. The studies available in the literature, and performed with accelerometers, did not use the same position to place the device. In our study, the accelerometer was placed on a semi-elastic belt covering the L4-L5 intervertebral space. Thus, our results cannot be completely comparable with other studies using different positions [29]. A standardized position of wearing the device is needed in the future. We acknowledge that process of data extraction could have represented another limiting factor. However, in our study, the accelerometer directly transmitted the data to a PC via Bluetooth. The sensitive axes of the sensing unit were automatically aligned along the anatomical vertical, medio-lateral, and antero-posterior axis. Finally, gold standard measurements of gait speed assessment, such as instrumented walkways and stereo photogrammetry [30], were not used in the present study.

Despite these limitations this is the first study having assessed the correlation of 4-MM with 4-MA. The association between these two techniques of gait speed assessment should be better investigated in larger populations, with longitudinal design, including also subjects with known dismobility syndrome. On this regard longitudinal studies comparing the two tests are needed to validate 4-MA as a gold standard. Meanwhile, 4-MM remains the easiest approach to be used in the clinical practice. The implementation of technological methods of gait speed assessment in second-level geriatric care services or for clinical research purposes should however be considered although based on our findings it becomes hard to make recommendations on the better usefulness of automatic than manual measurement.

## Conclusions

In a population of elderly community-dwellers without known dismobility syndrome, we found a significant correlation between the assessment of gait speed using manual and technological techniques. The concordance of gait speed manually or instrumentally assessed is not optimal and manual measures might lead to misclassification of a substantial number of subjects. However, there is need of future longitudinal studies comparing the two techniques, using standardized procedures and taking quantitative gait analysis as gold standard reference, before to recommend the usefulness of accelerometers in the comprehensive geriatric assessment.
